# A Handbook of Obstetric Nursing, for Nurses, Students and Mothers

**Published:** 1896-06

**Authors:** 


A HANDBOOK OF OBSTETRIC NURSING, for Nurses, Students and Mothers. By ANNA M. FULLERTON, M. D., Obstetrician, Gynecologist and Surgeon to the Woman's Hospital of Philadelphia; Clinical Professor of Gynecology to the Woman's Medical College of Pennsylvania. Fourth, revised, edition. Illustrated. Philadelphia: P. Blakiston, Son & Co., Publishers. 1895.

As indicated by the author, this book comprises "the course of instruction in obstetric nursing given to the pupils of the training school for nurses connected with the Woman's Hospital of Philadelphia." There are chapters on the pelvic and genital organs; signs, management and accidents of pregnancy; germs and antisepsis; application of antisepsis to confinement nursing; preparations for labor; signs of approaching labor and the process of labor; duties of the nurse during labor; accidents and emergencies of labor; management of the lying-in; care of the newborn infant; characteristics of infancy in health and disease and ailments of early infancy. This little work is thoroughly practical and admirably adapted as a text-book to be used in training schools for nurses, since the principles set forth are in accord with the "requirements of modern practice." It is an interesting volume, most valuable of its kind and deserves to be appreciated by those for whom it was written. E. B. W.

